# Teaching

**Published:** 1864-04

**Authors:** J. Foster Flagg


### Selections.

TEACHING. *By J. Foster Flagg, D. D. S.*—That it is no easy task to instruct, will, I think, be readily acceded when we recognize how few have made themselves acceptable to each one of us during that educational course which has been presented for our acceptance from the days of our childhood even to the present period of our existence.

That the requirements for instructing the youthful and adult mind are widely different, no one would for a moment deny; but the perception of ability for apprehension on the part of the student must be relatively the same: the child must have the mysteries of addition and substraction explained to him, as must the youth the beautiful demonstrations of Euclid; and that first lessons in geography and philosophy should be made advantageous to the juvenile seeker after knowledge, the information thus imparted should be placed before the understanding, and prepared for mental digestion with as much care and *tact* as would relatively be required to make the mature student clearly comprehend that which is known of the wonders of chemistry, physiology, and pathology.

In that the powers of mental prehension are different, so must the powers of impartation be different; but whether the subjects be simple or abstruse, the good results can only be obtained through the agency of a *forte,* the essence of which is instrinsically the same.

The work of education has often been denominated a "labor of love," and much has been said and written of the trials of the life of a teacher, but it seems to me that some argument might find place in this connection as to where the difficulty should be sought, for in this as in the successful practise of our profession, it is of the utmost importance that we search keenly for the *cause* of disease.

When we see that the task seems irksome, that the instructor labors, that those who would learn are inattentive, that the sitting seems long and the matter uninteresting, may we not well question as to why all this is so; and might we not very frequently find that the "trial" is rather on the part of the students in consequence of the fact that he who essays to teach has mistaken his vocation? Thinking, as he no doubt does, that he possesses sufficient knowledge of his subject to teach it, he is still in complete innocency of his total ignorance regarding the first principles of teaching.

It is a rare gift, that of preparing the common articles of animal food so as to make them acceptable to the general taste; how much more rare might we then reasonably suppose to be the quality of so preparing mental food as to render avidity concomitant with its acceptance! It is a consummation to be desired by many, hoped for by few, and realized only in those designed by nature and prepared by years of study and practice for the accomplishment of this great purpose. Is it to them a "labor of love," an irksome task, a daily trial? Indeed it is not. It is as the gushing forth from the surcharged scource of sparkling, cooling water, as the elimination of light and heat from the centre of a solar system, as the droppings from a sanctury.

Teachings fall pleasantly upon the ear from these, precepts are stored away in cerebral labyrinths, rugged paths in knowledge are shorn of their difficulties, and the crooked is made straight.

Such teachings should be cherished with nurturing care, protected by those who have the capacity to recognize them, and be stimulated to greater excellence through the endeavors of their recipents to bear fruit.—*Dental Cosmos.*

